# Triazole-Estradiol Analogs Induce Apoptosis and Inhibit EGFR and Its Downstream Pathways in Triple Negative Breast Cancer

**DOI:** 10.3390/molecules30030605

**Published:** 2025-01-30

**Authors:** Felix Acheampong, Trevor Ostlund, Emily Hedge, Jacqueline Laddusaw, Faez Alotaibi, Yaseen A. M. M. Elshaier, Fathi Halaweish

**Affiliations:** 1 Department of Preclinical Pharmacology and Toxicology, Verve Therapeutics Inc., Boston, MA 02215, USA; 2 Department of Chemistry and Biochemistry, College of Natural Sciences, South Dakota State University, Brookings, SD 57007, USA; ostlu104@umn.edu (T.O.);; 3 Department of Chemistry, College of Science, Qassim University, Buraydah 51452, Saudi Arabia; 4 Department of Organic and Medicinal Chemistry, University of Sadat City, Monufia 32897, Egypt

**Keywords:** epidermal growth factor receptor, triple negative breast cancer, triazole analogs, apoptosis, molecular dynamic simulations

## Abstract

Triple negative breast cancer, TNBC, is a difficult disease to treat due to relapse and resistance to known therapies. Epidermal growth factor receptor (EGFR), a tyrosine kinase responsible for downstream signaling leading to cell growth and survival, is typically overexpressed in TNBC. Our previous work has detailed the synthesis of triazole-estradiol derivatives as inhibitors of EGFR and downstream receptors, and this work continues that discussion by evaluating them in EGFR-dependent TNBC cell models MDA-MB-231 and MDA-MB-468. Compound Fz25 was cytotoxic against both MDA-MB-231 and MDA-MB-468 cell lines, yielding IC_50_ values of 8.12 ± 0.85 and 25.43 ± 3.68 µM, respectively. However, compounds Fz57 and Fz200 were potent against only MDA-MB-231 cells, generating IC_50_ values of 21.18 ± 0.23 and 10.86 ± 0.69 µM, respectively. Pathway analyses revealed that Fz25, Fz57 and Fz200 arrested the G_0_/G_1_ phase of the cell cycle and concomitantly suppressed cell cycle regulators, cyclin D_1_, cyclin E and Dyrk1B in MDA-MB-231 cells. Additionally, all compounds inhibited EGFR and its downstream signaling pathways—extracellular receptor kinase (ERK) and the mammalian target of rapamycin (mTOR)—in a dose-dependent manner. Furthermore, Fz25, Fz57 and Fz200 induced apoptosis in MDA-MB-231 cells by modulating morphological changes, including chromatin condensation, and attenuating the levels of cytochrome c, APAF1, caspases-3 and -9 as well as cleaved PARP. Of these compounds, only Fz25 showed overall satisfactory ADMET properties in silico. Similarly, Fz25 showed suitable binding parameters explored using molecular dynamic simulations in silico. These findings suggest that Fz25 warrants further preclinical and clinical investigations as a new generation of triazole congeners with significant potency in EFGR-dependent TNBC.

## 1. Introduction

Breast cancer continues to be the most common solid tumor that affects women and is the leading cause of cancer-associated mortality in females worldwide [1]. The heterogeneous nature of the disease leads to multiple subtypes; each with different treatment options. One of the hardest to treat is triple-negative breast cancer (TNBC). TNBC, clinically defined by the absence of estrogen receptor (ER), progesterone receptor (PR) and human epidermal growth factor receptor 2 (HER2) expression, forms about 20% of all breast cancer cases [2]. Its pattern of spread is distinct from that of non-TNBC; TNBC has a greater propensity for brain and lung metastases, and a lower prevalence of bone metastases [3]. TNBC has an aggressive behavior, and this is partly attributed to the aberrant expression of EGFR [4,5].

EGFR is a transmembrane tyrosine kinase receptor member of the HER family. Phosphorylation of the intracellular EGFR kinase domain activates multiple downstream signaling pathways including RAS/ERK and PI3K/Akt/mTOR cascades that lead to the transcriptional regulation of genes involved in cell proliferation, motility, and survival. The RAS/ERK pathway regulates gene expression, cell cycle regulation, and anti-apoptotic measures, and exists as a common cancer therapeutic target [6]. The PI3K/Akt/mTOR pathway regulates diverse cellular functions such as cell cycle progression and apoptosis whereas deregulation of its activity contributes to cell transformation [7]. Phosphorylated Akt inhibits apoptosis and promotes cell survival by the phosphorylation and inactivation of several target proteins including the pro-apoptotic protein Bcl-2, and the tumor suppressor p53, a regulator of Bcl-2 [8]. Apoptosis is mediated in cells through intrinsic and extrinsic pathways. Both pathways converge on the regulation of caspase-dependent proteolysis of thousands of cellular proteins, membrane blebbing and endo-nucleolytic cleavage of chromosomal DNA [9].

EGFR is considered a key clinical target for therapeutic intervention in TNBC and many other cancers. Anti-EGFR agents, such as small molecule tyrosine kinase inhibitors (TKIs) and monoclonal antibodies, are currently being investigated to treat TNBC [10]. However, only minimal success has been achieved to date, and therefore the need to discover new, more effective anti-TNBC agents is imperative. Currently, endogenous metabolites of mammalian origin have become an alternate source for the discovery of anticancer agents for therapeutic intervention. In this regard, estradiol analogs have turned out to be the focus of research due to their ability to exert significant antimitotic effects against various cancers including breast cancer. Estradiol is a highly fused polycyclic ring of estrogen origin with an aromatic ring A, two six membered rings B and C, and a cyclopentane ring D. Natural and synthetic molecules lacking an aromatic ring A have been reported to show no or weak bioactivity [11]. However, estradiol congeners possessing an aromatic ring A show potent bioactivity. In particular, Stander et al., 2011, reports that 2-methoxyestradiol (2ME), an endogenous metabolite of 17β-estradiol, and having an aromatic ring A, exhibits antiangiogenic and anti-breast cancer capabilities in vitro and in vivo [12]. Also, 17β-estradiol methyl ether (EDME) and its synthetic metabolites, PRU-10 and PRU-12—all containing aromatic ring at position A—act as inhibitors of TNBC cell migration and invasion [12]. These compounds exert their mechanistic effects independently of the cellular estrogen receptors and have no significant systemic hormonal effects [13]. Further examination of the structure–activity relationship revealed that a variation on ring D in addition to modifications at position 3 on aromatic ring A are important for the in vitro and in vivo potency of bioactive compounds and their synthetic analogs.

The findings from the above investigations imply that modifications of estradiol on ring A (position 3) and on ring D (position 17) are vital to generating lead candidates with improved potency and pharmacokinetic properties. Previously, our research group synthesized different estrone analogs by making modifications on positions 3 on ring A and 17 on ring D. Many of our estrone analogs, especially those bearing the cucurbitacin pharmacophores on position 17 on ring D, have demonstrated potent cytotoxic effects against distinct cancers in vitro [14,15,16,17]. Recently, our research group synthesized a new series of estradiol-1,2,3-triazole hybrids using pharmacophore-docking-based virtual screening. Hybrid compounds with 1,2,3-triazole as side chains or as scaffolds can act as multi-target single molecules and are cytotoxic against several cancers [18,19,20]. Additionally, hybrid compounds have captured an important position in cancer therapy, and hybridization of estrone scaffolds with 1,2,3-triazole pharmacophores may furnish valuable therapeutic intervention for cancer treatment.

Previously, we reported on the synthesis and cytotoxic effects of some estradiol-1,2,3-triazole analogs [20]. This study described some initial biological findings in which these analogs showed cytotoxic effects against both colorectal and ovarian cell cancer lines. Additionally, we described their effect against some EGFR pathway proteins, namely STAT3 and ERK, as well as ABC transporter proteins responsible for drug resistance. However, this original study lacked a true biological perspective describing their in vitro effects. This study helps bridge some information gaps in our previous study by highlighting the advanced biological effects of these analogs in triple negative breast cancer, namely the analysis of cell-cycle moderators, expanded EGFR-pathway protein analysis, apoptosis screening, molecular dynamic interactions, and ADMET factors for drug compatibility. Additional findings of this work not presented here can be found in Acheampong, 2019 [21].

## 2. Materials and Methods

### 2.1. Reagents and Chemicals

Antibodies against EGF Receptor (D38B1) XP^®^, Phospho-p44/42 MAPK (Erk1/2) (Thr202/Tyr204) (197G2), Cyclin D1 (92G2), mTOR, Phospho-mTOR (S2448), and anti-rabbit DyLight 680 conjugate (5366) secondary antibody were purchased from Cell Signaling Technology (Danvers, MA, USA); phospho-cyclin D1 (Thr286) (A537487) and anti-mouse DyLight 800 conjugate (W10815) from Thermo Fisher Scientific (Waltham, MA, USA); Phospho-EGF Receptor (Tyr1068), Dyrk1B, caspase-9, caspase-3, PARP1, cleaved PARP1, APAF1, cytochrome C, and GAPDH were purchased from Santa Cruz Biotechnology Inc. (Dallas, TX, USA). Phosphate buffered saline (PBS) and trypan blue solution were purchased from Thermo Fisher Scientific (Waltham, MA, USA). RealTime-Glo™ Annexin V apoptosis and necrosis assay (JA1011) kit were purchased from Promega (Madison, WI, USA). PI/RNase staining buffer was purchased from BD Biosciences (San Jose, CA, USA). Compound cytotoxicity was evaluated through measurement of mitochondrial dehydrogenase activities with 3-(4,5-dimethylthiazol-2-yl)-2,5-diphenyltetrazolium bromide (MTT) reagent (Sigma-Aldrich, St. Louis, MO, USA). The Hoechst 33342 stain was utilized for evaluating chromatin condensation and nuclear fragmentation, and was purchased from Thermo Fisher Scientific (Waltham, MA, USA). Sorafenib (positive control) (Selleckchem, Houston, TX, USA) and novel triazole-derived estradiol analogs were dissolved in dimethyl sulfoxide (DMSO) (Fisher Chemical/Fisher Scientific, Waltham, MA, USA). All other chemicals were of analytical grade.

### 2.2. Molecular Docking-Based Virtual Screening

**Figure 1:**
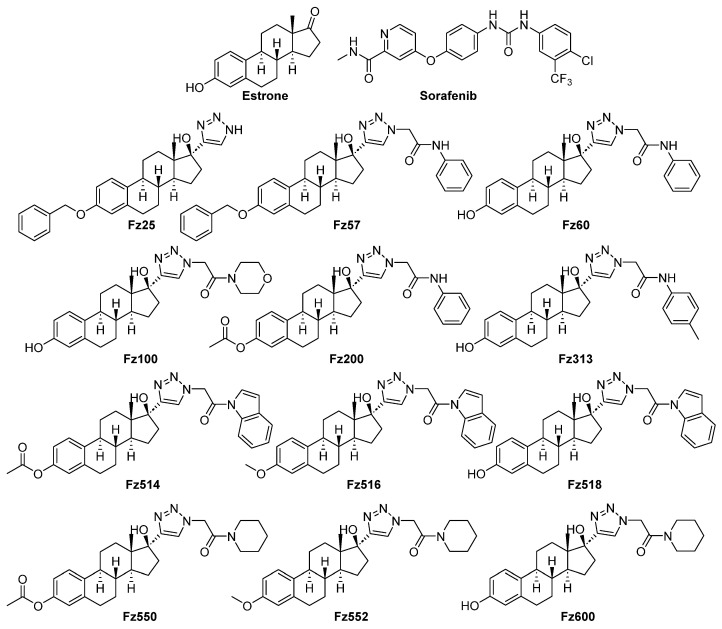
Molecular structures of estradiol-1,2,3-triazole analogs described in this study.

**Figure 2:**
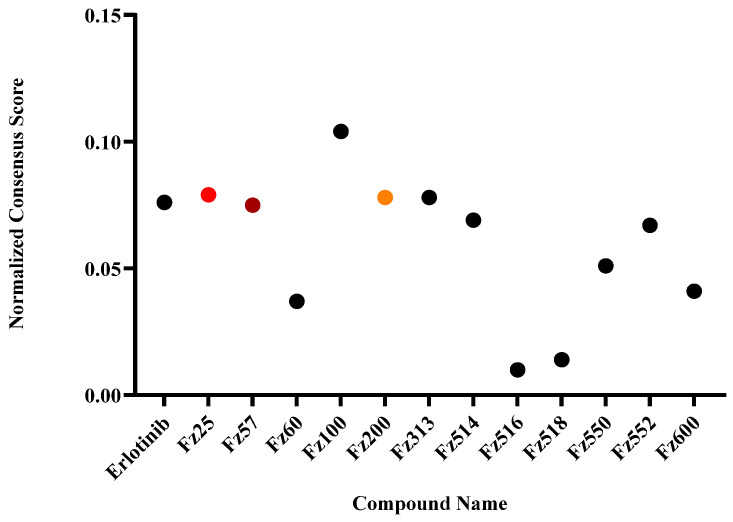
Molecular docking of designed estradiol-1,2,3-triazole analogs against EGFR binding site (Pdb: 1M17). Scatter plot of compounds normalized consensus score; consensus scores generated by VIDA application (Version 4.3.2). Light red: Fz25, Maroon: Fz57, and Yellow: Fz200.

Virtually designed molecules (Figure 1) were docked against EGFR protein (PDB ID: 1M17) target as previously described with slight modifications [15]. The OpenEye^®^ software package (Version 4.3.2) generates consensus scoring which is a filtering process to obtain virtual binding affinity. The consensus scores were normalized with compounds’ molecular weight to generate normalized consensus scores (Figure 2). The lower the normalized consensus score of a compound, the better binding affinity towards the EGFR protein target.

### 2.3. Cell Culture

MDA-MB-468 (a model for TNBC with high EGFR expressions) and MDA-MB-231 (a model for TNBC with moderate EGFR expressions) cell lines were purchased from American Type Culture Collection (ATCC, Manassas, VA, USA). The cells were cultured in Dulbecco’s Modified Eagle Medium (DMEM) supplemented with 10% (*v*/*v*) fetal bovine serum (FBS), 1% antibiotic/antimycotic (Gibco™, Thermo Fisher Scientific) at 37 °C equilibrated with 5% (*v*/*v*) CO_2_ in humidified air. The cells for the assays were detached using a solution of trypsin with 0.25% EDTA (Thermo Fisher Scientific).

### 2.4. Cytotoxicity Assay

The effect of triazole-estradiol analogs on cell viability was tested with MTT reagent as described with slight modifications [22]. Briefly, to measure mitochondrial dehydrogenase activities, cells were seeded in 96-well plates at an initial density of 30,000 cells per well. After overnight incubation, cells were treated with different concentrations of compounds in a dose range of 0–50 μM. The final DMSO concentration was 0.05%. After 48 h of incubation, 20 μL of MTT reagent (5 mg/mL) was added to each well and the resulting formazan crystals were dissolved in 250 μL of dimethyl sulfoxide (DMSO). Four independent experiments were completed to determine the mean optical density referred to as cell viability, using Hidex Sense Beta Plus plate reader (Turku, Finland). Cell viability was expressed as a percentage of DMSO-treated controls.

### 2.5. Flow Cytometry for Cell Cycle Analysis

MDA-MB-231 and MDA-MB-468 cells were seeded into six-well plates at a concentration of 300,000 cells per well and allowed to attach in culture overnight, then treated with IC_50_ of compounds for 48 h. Afterward, cells were washed with PBS and harvested. Cell cycle analysis was investigated by adding propidium iodide (PI) stain (Thermo Fisher Scientific) to 1 mL of cell suspension. Briefly, harvested cells were fixed in 70% ethanol and incubated at 4 °C for 4 h. Subsequently, the cells were cleaned of alcohol and stained with RNase free PI solution. The cell suspension was incubated in the dark for 45 min at room temperature. The samples were analyzed by flow cytometry and compared to DMSO-treated cells. These experiments were performed on a BD Accuri™ C6 flow cytometer (BD Biosciences, San Jose, CA, USA) using BD Accuri™ C6 software, version 1.0.

### 2.6. Annexin V Assay

The Annexin V assay was performed in accordance with the manufacturer’s protocol with slight modifications. Briefly, MDA-MB-231 cells were seeded in wells on a 96-well white plate with clear bottom at an initial density of 30,000 cells per well for overnight attachment. Afterward, the cells were incubated with the IC_50_ of compounds or camptothecin control for 6 h. Subsequently, the RealTime-Glo™ Annexin V Apoptosis reagent (Promega, Madison, WI, USA) was prepared (sequentially mix Annexin NanoBiT^®^ Substrate, CaCl_2_, Annexin V-SmBiT and Annexin V-LgBiT in a prewarmed media) and added to the reaction set up before incubating for additional 1 h. Luminescence was measured afterward using Hidex Sense Beta Plus plate reader (Turku, Finland). Apoptosis was expressed as a percentage relative to DMSO-treated controls.

### 2.7. Morphological Analysis with Fluorescence Microscopy

To evaluate the apoptotic activity of the analogs, nuclear staining was performed with the DNA-binding dye Hoechst-33342 in accordance with manufacturer’s protocol. In brief, MDA-MB-231 cells were plated into 6-well plates and treated with IC_50_ of compounds or sorafenib standard for 24 h. Cells were washed with PBS and incubated with Hoechst-33342 (10 μg/mL) for 15 min in the dark, then observed under a fluorescence microscope (excitation 352 nm, emission 461 nm; NIKON TE2000-E, Nikon Instruments Inc., Melville, NY, USA). Apoptotic cells were identified by condensation of chromatin and fragmentation of nuclei. Pictures were obtained using a video camera Q-imaging system (Burnaby, BC, Canada).

### 2.8. In-Cell Western (ICW) Assay

Proteins involved in apoptosis, cell cycle progression, and EGFR pathways were quantified by ICW. This technique was carried out in accordance with the manufacturer’s instructions with slight modifications. Briefly, about 50,000 cells per well were seeded into 96-well black walled plate with clear bottom for overnight attachment. Cells were then treated with IC_50_ compounds for 24 h. Subsequently, cells were fixed with 3.7% formaldehyde solution, permeabilized with 0.1% Triton X-100 solution and blocked with fish gel buffer (1×) prior to primary antibody addition. Wells were then incubated with the relevant antibodies: EGF Receptor (D38B1) XP^®^, Phospho-p44/42 MAPK (Erk1/2) (Thr202/Tyr204) (197G2), Cyclin D1 (92G2), phospho-cyclin D1 (Thr286) (A537487), mTOR, Phospho-mTOR (S2448), Phospho-EGF Receptor (Tyr1068), Dyrk1B, caspase-9, caspase-3, PARP1, cleaved PARP1, APAF1, cytochrome C, and GAPDH control overnight followed by an hour incubation with anti-mouse or anti-rabbit DyLight conjugated (680 or 800 nm) secondary antibodies. Images were acquired using LICOR Odyssey^®^ Fc Imaging System (Lincoln, NE, USA) and image quantification carried out by Fiji software (Image J, Java 1.8.0). An example of ICW assay data is seen in Supplemental Figure S1.

### 2.9. Evaluation of Physicochemical, Drug-Likeness and ADMET Properties of Estradiol-1,2,3-triazole Hybrids

Physicochemical and ADMET properties of estradiol-1,2,3-triazole hybrids were estimated based on previous reports with some modifications [23]. Physicochemical properties of the estradiol-1,2,3-triazole hybrids were estimated using ADMETLab 2.0 and SwissADME webservers (Version 1.0) [24,25]. ADMETLab 2.0 and SwissADME are free web tools to evaluate pharmacokinetics, drug-likeness, and medicinal chemistry acceptability of small molecules. The estimated characteristics with SwissADME included HBA (hydrogen bond acceptor), MW (molecular weight), MlogP (octanol/water partition coefficient), HBD (hydrogen bond donor), RB (rotatable bond count), and TPSA (topological polar surface area). According to Lipinski’s rule of five (RO5), the orally active compounds should contain no more than 5 HBD and 10 HBA. In addition, Veber’s rule suggested that compounds which meet only the two criteria of 10 or fewer rotatable bonds and polar surface area equal to or less than 140 Å^2^ have a high probability of good oral bioavailability in rats. Furthermore, MlogP, MW, and TPSA of the orally bioavailable compounds should be less than 5, 500, and 140 Å2, respectively. The percentage ABS was computed as follows: %ABS = 109 − [0.345 × TPSA] [26]. LogD_7_._4_ (octanol/water partition coefficient at pH 7.4) and PAINS alerts were computed with ADMETLab 2.0.

All ADMET properties except GI absorption and Abbot bioavailability were predicted with ADMETLab 2.0 webserver [27] from the compounds’ Simplified Molecular Input Line Entry System (SMILES) string as input. Potentially toxic compounds that were AMES mutagens, carcinogens, and human Ether-a-go-go-Related gene (hERG) inhibitors will be totally discarded from further studies. The inhibition of the human ether-a-go-go (hERG) ion channel may cause cardiotoxicity and hence, it is necessary to screen all potential drug candidates for risk against blocking the hERG channel [23]. The AMES test is a sensitive tool in screening for potential genotoxic carcinogens.

### 2.10. Molecular Dynamic Simulations

Analog Fz25 underwent dynamic simulation in two proteins investigated in the biological study, EGFR and ERK (PDB codes 1M17 and 2ZOQ), to determine important pharmacophores necessary for biological activity. Procedural steps were performed as reported in [15]. Molecular dynamic simulations were then performed using Schrodinger Desmond Maestro software (Release 2023-1) [28].

### 2.11. Statistical Analysis

Microsoft^®^ Excel^®^ for Windows, version 16.0., was used for the calculation of mean and SD values of different experiments. Plotting of bar graphs was carried out using GraphPad Prism 5.01 (San Diego, CA, USA). Mean IC_50_ values were compared by one-way ANOVA and multiple comparisons were made by the Dunnett Test using GraphPad Prism 5.01 (San Diego, CA, USA) and values with *p* < 0.01 or *p* < 0.001 were considered statistically significant. Cell cycle analysis and protein expression assays were deemed significant by comparing the untreated DMSO control and analogs using a two-way ANOVA with Bonferroni correction. *p*-values < 0.05 were considered statistically significant.

## 3. Results

### 3.1. Triazole-Estradiol Analogs Exhibit Cytotoxic Effects Against MDA-MB-231 and MDA-MB-468 Cells

**Figure 3:**
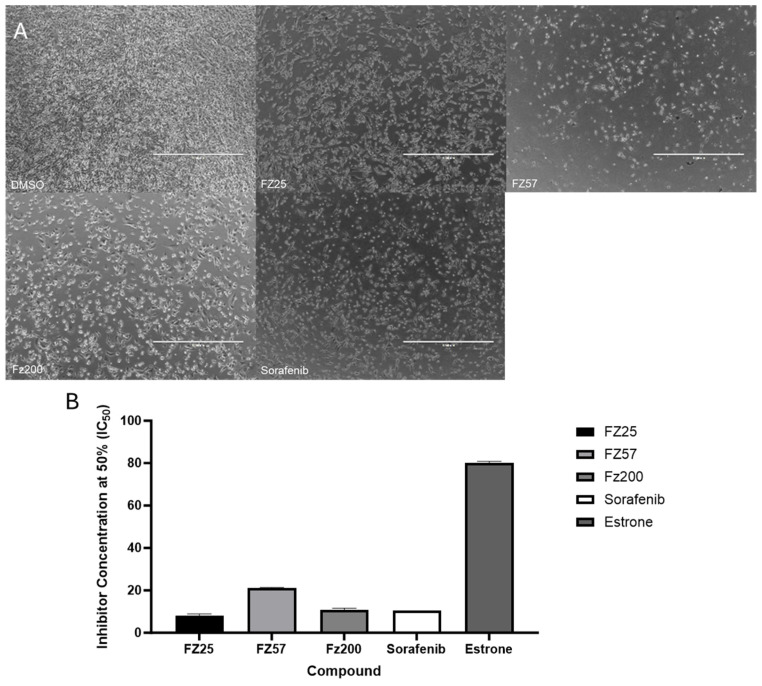
(**A**) Morphological changes in MDA-MB-231 cells after treatment with 0.05% DMSO, Fz25, Fz57, Fz200 and sorafenib. Images were acquired with ×4 objective lens of Evos XL cell imaging system (Thermo Fisher Scientific), scale bar = 1000 µm. (**B**) Cytotoxicity assay of triazole-estradiol analogs against MDA-MB-231 cell line within 48 h of incubation. Data are representative as a mean IC_50_ ± SEM of four independent experiments.

To evaluate the cytotoxic effects of the estradiol analogs against MDA-MB-231 and MDA-MB-468 cells, the cells were initially treated with different concentrations of the compounds between 0 and 50 μM. Effects on cell viability were determined by the MTT assay after 48 h. Overall, some compounds did show a significant cytotoxic effect toward both TNBC cell lines within the dose range tested after 48 h (Table 1). Particularly, we observed that Fz25, Fz57, Fz200, Fz300 and Fz400 recorded IC_50_ values of 8.12 ± 0.85, 21.18 ± 0.23, 10.86 ± 0.69, 14.95 ± 1.85 and 21.21 ± 0.76 μM, respectively, against MDA-MB-231 cells as compared to sorafenib control of 10.62 ± 0.02 μM. Morphological changes by the analogs in the MDA-MB-231 cell line (Figure 3) were quite apparent for cytotoxicity as well. We saw less bioactivity in the MDA-MB-468 cell line; however, Fz25 recorded an IC_50_ of 25.43 ± 3.68 µM as compared to sorafenib of 28.38 ± 3.13 µM, suggesting it likely plays a role acting against TNBC.

### 3.2. Fz25 Inhibits the Proliferation of MDA-MB-231 Cells Through G_1_ Phase Cell Cycle Arrest

**Figure 4:**
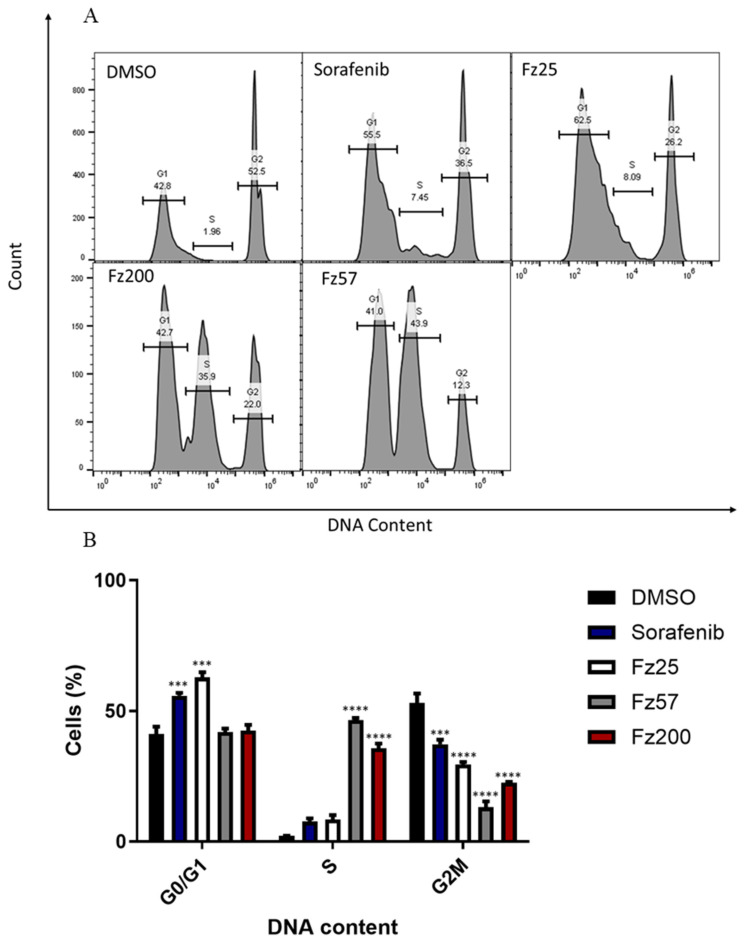
Estrone analogs exposure resulted in G1- or S-phase cell cycle arrest. MDA-MB-231 cells were treated with IC_50_ values of estrone analogs and analyzed by flow cytometry after 48 h. (**A**) Distribution of cells in distinct phases of the cell cycle. Fz25 and sorafenib, positive control showed G_0_/G_1_ phase cell cycle arrest compared to negative control, DMSO, as obtained by flow cytometry. Fz57 and Fz200 showed S-phase cell cycle arrest. (**B**) The bar graphs show mean ± SD of the percentages of MDA-MB-231 cells in the different phases of the cell cycle (G_0_/G_1_, S and G_2_/M). At least three independent experiments were performed. *** *p* < 0.001, **** *p* < 0.0001 significant differences in cell cycle arrest compared to DMSO control.

The quantification of cells at various stages within the cell cycle was determined with propidium iodide stain and analyzed by flow cytometry. Cell cycle distribution of MDA-MB-231 cells exposed to DMSO (Figure 4) showed an average of 41.2% in the G_0_/G_1_ phase, 2.2% in the S phase and 53.2% in the G2/M phase, respectively. The exposure of cells to Fz25 resulted in a significant accumulation of cells within the G_0_/G_1_ phase in the MDA-MB-231 cell line, an average of 62.8%; there was also a decreased enrichment of cells in the G_2_/M phase, an average of 29.5% as a result. Additionally, Fz57 and Fz200 showed a significant S phase arrest, suggesting their pathway of inhibition may be slightly different. In the MDA-MB-468 cell line, Fz25 showed a G_0_/G_1_ phase accumulation of around 39% and a G_2_/M accumulation of 37%, not significantly different from the initial DMSO experiment control.

### 3.3. G_1_ Phase Cell Cycle-Associated Proteins Are Modulated in MDA-MB-231 Cells After Exposure to Fz25

**Figure 5:**
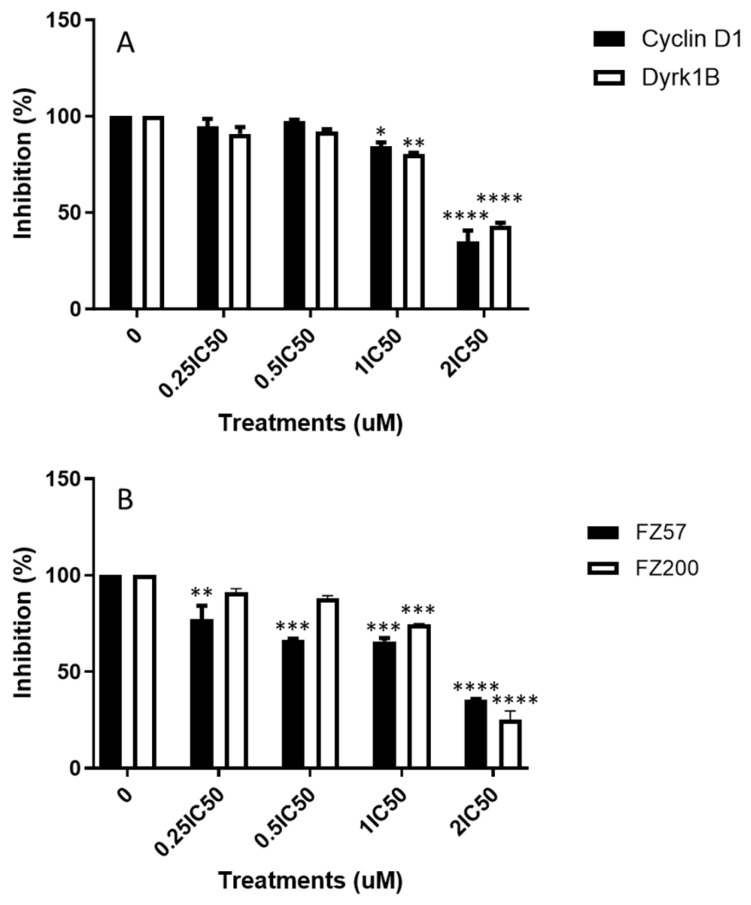
Effects on G_1_- or S-phase cell cycle regulators after treatment of MDA-MB-231 cells with estradiol analogs. Protein quantification was completed using scanned images from Fiji software, and expression levels of the proteins normalized to GAPDH. Bar graphs are means ± SD from at least two independent experiments. (**A**) Fz25 and (**B**) Cyclin E expression inhibited by Fz57 and Fz200. Significance levels of * *p* < 0.05, ** *p* < 0.01, *** *p* < 0.001, **** *p* < 0.0001.

As a result of the significant (*p* < 0.001) cell cycle arrest induced by Fz25 in the MDA-MB-231 cell line, we analyzed the expression levels of known G1 phase cell-cycle proteins. The in-cell Western approach was adopted to establish our findings. Cyclin D1 and Dyrk1B play important roles in regulating the progression of the cell cycle within the G1 phase. We observed that Fz25 administration to MDA-MB-231 cells resulted in the suppression of cyclin D1 and Dyrk1B in a concentration-dependent manner within 24 h (Figure 5A), suggesting that Fz25 is modulating their activity.

### 3.4. EGFR and Its Downstream Signaling Pathways Are Inhibited After Exposure of Fz25, Fz57 to TNBC Cells

**Figure 6:**
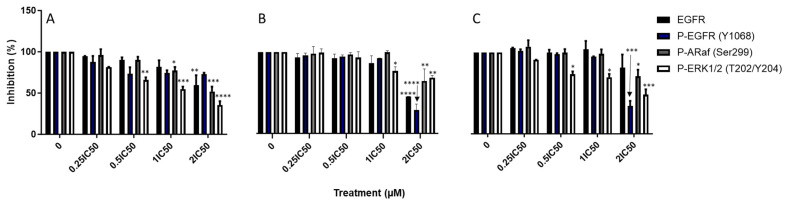
Effect of triazole-estradiol analog treatment on EGFR and downstream ERK1/2 effector molecules in MDA-MB-231 cells. Protein quantification was completed using scanned images from Fiji software, and expression levels of the proteins normalized to GAPDH. Bar graphs are means ± SD from at least two independent experiments. (**A**) Fz25, (**B**) Fz57, and (**C**) Fz200. Significance levels of * *p* < 0.05, ** *p* < 0.01, *** *p* < 0.001, **** *p* < 0.0001.

**Figure 7:**
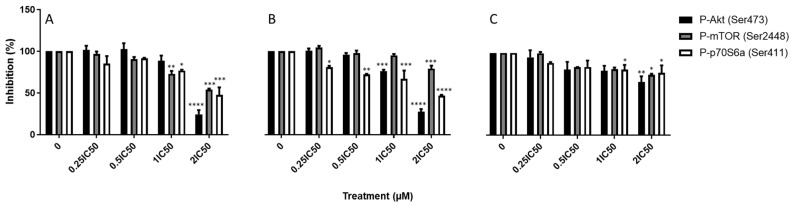
Effect of triazole-estradiol analog treatment on Akt pathway proteins in MDA-MB-231 cells. Protein quantification was completed using scanned images from Fiji software, and expression levels of the proteins normalized to GAPDH. Bar graphs are means ± SD from at least two independent experiments. (**A**) Fz25, (**B**) Fz57, and (**C**) Fz200. Significance levels of * *p* < 0.05, ** *p* < 0.01, ***p < 0.001, **** *p* < 0.0001.

We evaluated the expression levels of total and activated (phosphorylated) forms of EGFR by ICW as well as downstream signaling proteins in the PI3K/AKT/mTOR and RAS/ERK pathways (Figure 6 and Figure 7). Higher levels of EGFR were detected in the DMSO-treated cells compared to compound treated cells, depicting EGFR downregulation in MDA-MB-231 cells. Similarly, levels of phosphorylated EGFR (Y1068) were downregulated in a concentration-dependent manner as opposed to DMSO treated cells. The purpose of EGFR autophosphorylation is to activate signaling pathways, such as PI3K/AKT/mTOR and RAS/ERK pathways [29]. We next investigated the activation of these pathways by quantifying the activated (phosphorylated) forms of various downstream proteins in each pathway. Decreased amounts of phospho-ERK1/2 (T202/Y204) were detected when exposed to Fz25, Fz57 and Fz200 in a dose-dependent manner (Figure 6). Also, phospho-mTOR (S2448), phospho-Akt (S473), and phosphor-p70S6a (S411) levels were downregulated in a concentration-dependent manner as well after exposure to Fz25 and Fz57 (Figure 7). Based on the levels of downstream protein phosphorylation, we can conclude the cytotoxic triazole analogs have some effect on EGFR and downstream signaling proteins in both TNBC cell lines studied. Due to inherent nonspecificity of some antibodies used in the ICW analysis, further validation on protein levels are likely required for future work in this area.

### 3.5. Apoptosis Markers in MDA-MB-231 Cells

As we saw similar results at this point in both TNBC cell lines as well as in previously tested cancer cell lines [20], we wished to explore how these triazole-estradiol analogs acted on proteins associated with apoptosis in a TNBC model. To do this, we explored typical expression markers seen in apoptosis, such as phosphatidylserine (PS) expression on the outside of cells, cellular morphology changes, and activation of caspases and other apoptotic protein markers in MDA-MB-231 cells.

### 3.6. RealTime-Glo™ Annexin V Assay Details Increased Apoptosis by Fz25, Fz57, and Fz200

**Figure 8:**
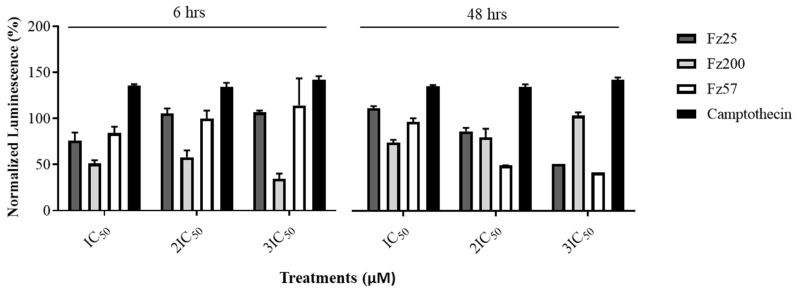
Initial apoptosis induction in MDA-MB-231 cells assayed by Annexin V after 6 h and 48 h of incubation with drug treatment. Cells were treated with IC_50_, 2IC_50_ and 3IC_50_ of estradiol analogs or positive control, camptothecin. The cells were then incubated with Annexin V reagent and luminescence measured by a luminometer. At least two independent experiments were performed in triplicate.

The cell death inducing effect of the potent triazole-estradiol analogs in MDA-MB-231 cells were evaluated by RealTime-Glo™ Annexin V apoptosis and necrosis luminescent assay. The detection reagent contains Annexin V-LgBiT and Annexin V-SmBiT (NanoBiT) fusion proteins and a profluorescent DNA dye. In healthy cells, most of the phosphatidylserine (PS) is confined to the inner leaflets of the cell membrane; fewer fusion proteins bind to PS and less luminescence is recorded. In apoptotic cells, PS is exposed to the outer leaflet of the cell membrane, more fusion proteins bind to PS and increased luminescence is measured. Increased luminescence corresponds to increased apoptosis. Treating MDA-MB-231 cells with the analogs saw increased apoptosis observed in a dose-dependent manner for Fz25, Fz57, and Fz200 after 48 h (Figure 8). Camptothecin, a topoisomerase inhibitor that leads to apoptosis when administered, was used as a positive control. To normalize luminescence, the triazole-estradiol analog-treated cells’ luminescence was compared to the 0.05% DMSO-treated control cells’ luminescence and expressed as a percentage; this accounts for variations in cell number and overall assay conditions across experiments. The higher % normalized luminescence is indicative of higher apoptosis induction.

### 3.7. Morphological Analysis with Fluorescence Microscopy Reveals Fz25 Alters the Cellular Shape of TNBC Consistent with Apoptosis

**Figure 9:**
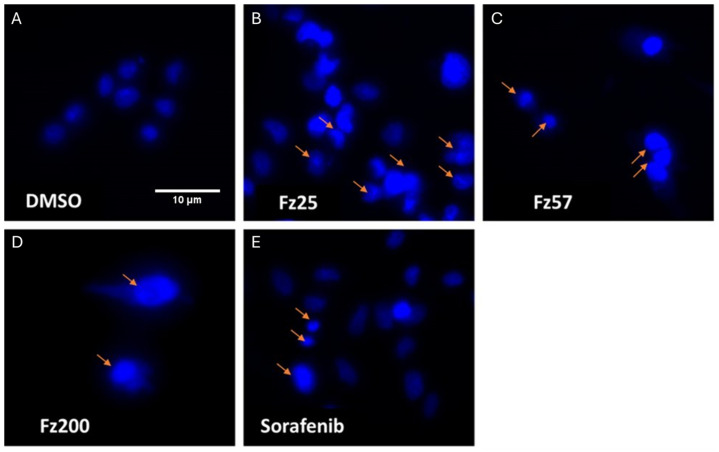
Apoptotic cells observed by Hoechst 33342 staining. MDA-MB-231 cells were treated with 0.05% DMSO (**A**), IC_50_ of estradiol analogs ((**B**) Fz25, (**C**) Fz57 and (**D**) Fz200) or positive control, sorafenib (**E**). The abnormal morphology of the condensed nuclei is indicated by orange arrows.

The morphological observation in the cell nuclei of MDA-MB-231 cells for 24 h after treatment with Fz25, Fz57, Fz200, or sorafenib showed significant morphological alterations when compared to 0.05% DMSO control. As shown in Figure 9, the control or untreated cells appeared to be intact oval shapes and the nuclei were stained with a less bright blue fluorescence (due to the Hoechst 33342 dye). Cells treated with triazole-estradiol analogs or sorafenib exhibited typical features of apoptosis such as cell shrinkage, chromatin condensation, and fragmentation in multiple, segregated bodies, the formation of apoptotic bodies, and cell decrement. The apoptotic nuclei clearly showed highly condensed or fragmented chromatin that was uniformly fluorescent. Drug treatments resulted in increased apoptotic cells (condensed chromatin, indicated by arrows) compared to the DMSO control.

### 3.8. Triazole-Estradiol Analogs Modulate Apoptotic Protein Factors from the Mitochondrial Apoptosis Pathway

**Figure 10:**
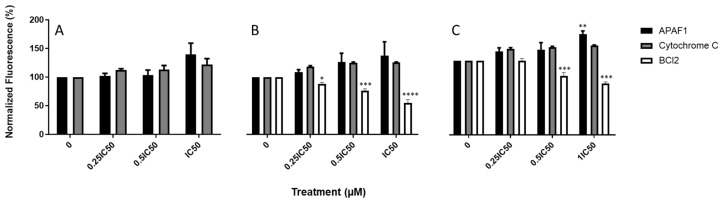
Expression levels of cytosolic cytochrome C and APAF1 in MDA-MB-231 cells assayed by in-cell Western (ICW). Quantitation of proteins was completed using scanned images from Fiji software, and expression levels of APAF1 and cytochrome C normalized to GAPDH. At least two independent experiments were performed. (**A**) Fz25, (**B**) Fz57, and (**C**) Fz200. Significance levels of * *p* < 0.05, ** *p* < 0.01, *** *p* < 0.001, **** *p* < 0.0001.

**Figure 11:**
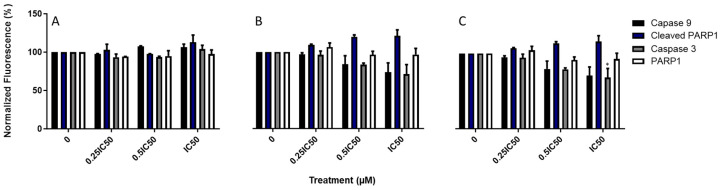
In-cell Western quantification of apoptosis-associated proteins. Quantitation of proteins was completed using scanned images from Fiji software, and expression levels of the proteins normalized to GAPDH. At least two independent experiments were performed. (**A**) Fz25, (**B**) Fz57, and (**C**) Fz200.

To determine whether the compounds modulated proteins involved in cell death, ICW assay was performed to detect changes in protein expression in the mitochondrial apoptosis pathway. As shown in Figure 10, the exposure of MDA-MB-231 cells to Fz25, Fz57, and Fz200 resulted in a presumed increase in the expression of cytochrome C and apoptotic protease activating factor 1 (APAF1), as well as a decrease in Bcl-2, which are markers of mitochondrial apoptosis. Also, Fz25 when administered to cells resulted in a slightly increased expression of cleaved PARP1 but had no effect on PARP1 in a dose-dependent fashion (Figure 11A). An insignificant increase in the expression levels of caspases-3 and -9 were also noted when MDA-MB-231 cells were exposed to Fz25, but this cannot be solely explained by the results in this assay.

### 3.9. Drug-Likeness Features and ADMET Properties

The efficacy of medicinal compounds is considerably dependent on the molecular traits and bioactivity of the drug candidates. To anticipate the drug-like features and bioactivity of the estradiol-1,2,3-triazole hybrids as EGFR inhibitors in TNBC, SwissADME and ADMETLab 2.0 webservers were utilized. Due to the large amount of data, the results are shown in full in the Supplemental Information. The estimated physiochemical characteristics are depicted in Table S1. Some compounds demonstrated violations in a few features like molecular weight (MW) and Lipinski’s rule. The MlogP and LogD_7.4_ of molecules were auspicious, with values ≤ 5. The TPSA values of all synthesized estradiol-1,2,3-triazole analogs were less than 140 Å^2^ and the number of hydrogen bond donors (HBD) was ≤5. The number of hydrogen bond acceptors (HBA) was ≤10. No PAINS alerts violation was detected for any molecule. Fz25 demonstrated suitably high acceptable gastrointestinal absorption (GIA), but the rest of the compounds depicted moderately acceptable GIA (Table S2). In addition, all molecules published satisfactory Caco-2 and MDCK permeability, Abbot bioavailability, half-life, clearance, and blood–brain-barrier profiles (Table S2). Fz100, Fz514, Fz550, Fz552 and Fz600 showed acceptable plasma protein binding ≤ 95. However, only Fz100, Fz550 and Fz600 depicted a high % fraction of unbound drug (greater than 5%). Most triazole-estradiol analogs were either substrates or inhibitors of P-glycoprotein, but Fz25, like sorafenib (positive control), was neither a substrate nor inhibitor of P-glycoprotein (Table S3). Also, almost all compounds were inhibitors of cytochrome P450 isoforms, via CYP1A2, CYP2C19, CYP2C9, CYP2D6 and CYP3A4. Fz25 showed acceptable non-toxic properties, having a *p*-value ≤ 3 for human hepatotoxicity, drug-induced liver injury (DILI), AMES toxicity, carcinogenicity, and eye irritation (Table S4). All compounds except Fz100 were moderate hERG blockers having *p*-value ≥ 0.3. However, we decided to perform further optimizations for hit compounds that had *p*-value < 0.9 because molecules with *p*-value > 0.9 are extremely toxic towards the hERG ion channel. For parameters like Pgp-inhibitor, hERG blocker, etc., predicted by the ADMETLab 2.0 models, these probability values are considered: 0–0.1, 0.1–0.3, 0.3–0.5, 0.5–0.7, 0.7–0.9, and 0.9–1.0. Usually, the token ‘0.9–1.0’ represents that the molecule is more likely to be toxic, while ‘0.0–0.1 and 0.1–0.3’ represents nontoxic molecules [25].

### 3.10. Molecular Dynamic Simulations Identify Key Pharmacophores of Fz25 Important for Analog Design

**Figure 12:**
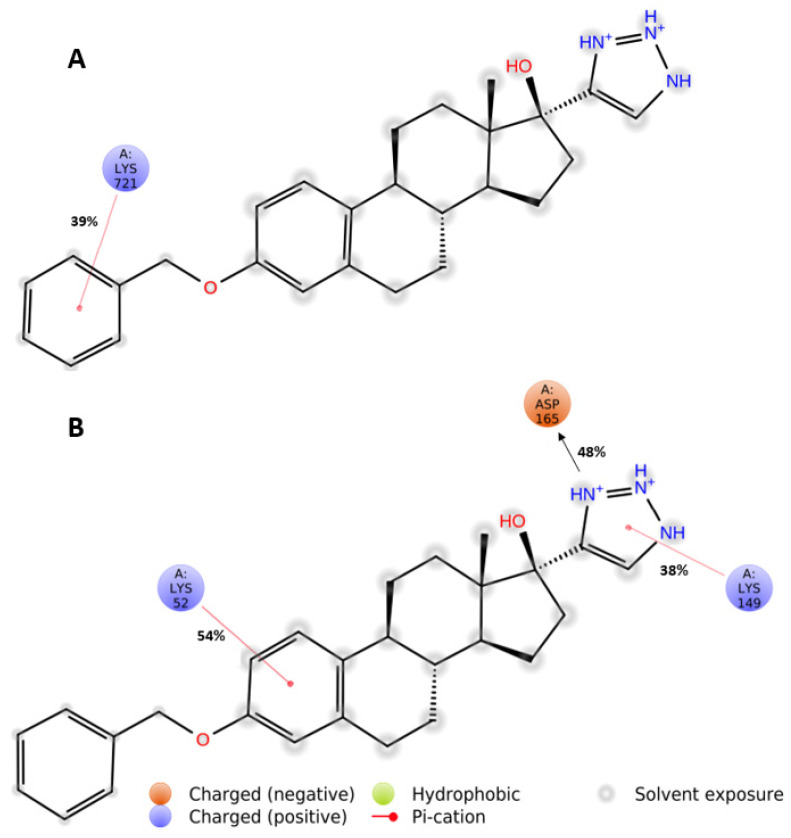
Molecular dynamic simulation results of ligand–protein contacts for Fz25 with (**A**) EGFR and (**B**) ERK. Direct arrows are hydrogen bonds/electrostatic interactions while red lines are cation–pi interactions. Percentages shown are the amount of simulation time relative to the entire simulation in which the contact between ligand and protein are maintained.

Molecular dynamic simulations were performed on Fz25 with both EGFR and ERK in the EGFR cascade. Ligand–protein contact diagrams (Figure 12) reveal that aromatic functionalities of the triazole analogs are key for important binding parameters to proteins inhibited by these analogs. Specifically, we see cation–pi interactions occurring with the C3 benzyl moiety during 39% of the simulation with Lys-721. In ERK, we see two more additional cation–pi interactions: one with the aromatic ring A to Lys-52 and one with the triazole group to Lys-149. These interactions occur in nearly half of the simulation, suggesting they play an important role in the binding of these analogs.

## 4. Discussion

Some 17β-estradiol derivates have been proven to be cytotoxic against various cancers including EGFR-dependent TNBC [11,30,31]. Mechanistically, the observed cytotoxicity has been attributed to the inhibition of angiogenesis, actin depolymerization, autophagy induction, reduced cell migration and invasion, and the modulation of both intrinsic and extrinsic apoptosis. However, only a few studies have investigated estrone or estrogen analogs’ inhibitory effects against EGFR and its downstream signaling pathways in TNBC [15,27,32]. EGFR is known to be moderately or highly expressed in TNBC, yielding several phenotypes. Previously, our research group has reported on diverse estrone analogs’ modulatory effects in different cancers dependent on EGFR expression [14,15,20,33]. Recently, we synthesized a new series of estradiol analogs (bearing substituted 1,2,3-triazole at position 17 on the D ring of the estrone scaffold) using pharmacophore-docking-based virtual screening (see Ref. [20] for more information). In this study, we report for the first time that some of the estradiol-1,2,3-triazole hybrids were strongly cytotoxic against MDA-MB-231 and weakly cytotoxic against MDA-MB-468 cell lines. Also, the bioactive compounds mechanistically elicited apoptotic induction and suppressed EGFR expression, as well as downregulating EGFR downstream signaling proteins ERK1/2, mTOR, and Akt.

To explore estradiol-1,2,3-triazole hybrids as potential EGFR inhibitors, the OpenEye^®^ molecular docking tool was first utilized to screen for in silico hit compounds against EGFR. A compound library was designed, energy minimized and virtually screened against EGFR with fast docking parameters. The consensus scores generated from the docking application were normalized with the molecular weight of each designed analog allowing for an effective comparison of binding affinity between low and high molecular weight compounds. According to the anticipated docking consensus scores, only one compound unveiled normalized consensus scores higher than the co-crystalized inhibitor (Erlotinib = 0.078) (Figure 2).

To corroborate the in silico findings, we assessed all the synthesized analogs in a high throughput screening (HTS) MTT assay against TNBC cells. Compounds with IC_50_ values ≤ 25 µM were promoted for further mechanistic studies in TNBC cells. Fz25, Fz57 and Fz200 showed IC_50_ concentrations of 8.12 ± 0.85, 21.18 ± 0.23, 10.86 ± 0.69 μM, respectively, compared to sorafenib, IC_50_ value of 10.62 ± 0.02 μM (Table 1) in MDA-MB-231 cells, confirming their cytotoxicity in vitro. Similarly, Fz25 was cytotoxic against MDA-MB-468 cell line with an IC_50_ of 25.43 ± 3.68 µM, comparable to sorafenib with an IC_50_ of 28.38 ± 3.13 µM. Fz57 and Fz200 were not potent against MDA-MB-468 cells. These findings suggest that the estradiol-1,2,3-hybrids are very sensitive to TNBC cells expressing low levels of EGFR, as MDA-MB-231 cells show low expressions of EGFR compared with MDA-MB-468 cells. Fz25 was designed and synthesized by introducing a phenoxy group on aromatic ring A (position 3) and 1,2,3-triazole on ring D (position 17). Other modifications like methoxy and hydroxy substituents were made on the third position of ring A, and N-phenyl acetamide, *p*-methyl-N-phenyl acetamide, etc., were made at position 17 on ring D to yield the rest of the triazole-estradiol congeners. The structure–activity relationships (SAR) revealed that compounds having pharmacophores like phenoxy or acetoxy groups introduced onto estrone’s aromatic A ring at position 3 and 1,2,3-triazole or N-phenyl acetamide-2,3-triazole substituents at position 17 on ring D are vital for triazole-estradiol analog bioactivity. Previously, we have reported that estrone hybrids containing pharmacophores including sulfamoyl or methoxy substituents on ring A (position 3) and *p*-nitrophenyl on ring D (position 17) were potent against MDA-MB-468 (TNBC cells with high EGFR expressions) [15]. Also, Rühl et al. 2021, reported that EDME and its novel congeners (modifications carried out on aromatic ring A at position 3) reduced migration and invasion in MDA-MB-231, corroborating their efficacy to interfere with cancer hallmarks in a TRPML1-dependent manner [11]. In a parallel study (unpublished data) conducted by our research group, we observed that Fz25 (IC_50_ of 8.13 ± 0.15) and Fz57 (IC_50_ of 11.94 ± 0.19) were cytotoxic against MCF-7 (ER + and EGFR -) breast cancer cells comparable to sorafenib, positive control (IC_50_ of 12.21 ± 0.96). These findings further demonstrate that the novel triazole-estradiol hybrids can target both EGFR-dependent and -independent breast cancer cells, suggesting that these agents can be further developed as multi-target single agents. We report here for the first time that introducing 1,2,3-triazole or modified 1,2,3-triazole substituents onto the D ring (position 17) and phenoxy or acetoxy onto aromatic A ring (position 3) of the estrone scaffold is important in sensitizing TNBC cells with moderate EGFR expressions.

Bioactive compounds exhibit cytotoxic killing in EGFR-dependent cancer cells through several mechanisms including: 1. Inhibition of cell proliferation and 2. Modulation of cell death [34]. EGFR signaling is one of the proliferation mechanisms that is widely studied in various cancers including TNBC. Upregulated activity of EGFR is an important molecular characteristic that has been noted in TNBC [35]. Activated EGFR affects four major signaling pathways: MAPK, PI3K/AKT/mTOR, PLCγ/PKC, and the JAK/STAT pathway [36]. MAPK activation leads to the modulation of target proteins, including cell cycle activator cyclin D1, regulating the growth and proliferation of cells [37]. Similarly, activated AKT exerts its survival and protein synthesis activities by directly activating the mammalian target of rapamycin (mTOR) and ribosomal protein S6 kinase (p70S6K) [38]. Until now, only two main classes of inhibitors have been discovered to target EGFR: monoclonal antibodies (mAbs) and TKIs. mAbs bind to the extracellular domain (ECD) of EGFR, and block ligand-receptor binding and consequently abrogating EGFR dimerization. TKIs act within the intracellular tyrosine kinase domain of EGFR, where they compete with ATP to eliminate EGFR downstream signaling. Despite numerous advances to discover anti-EGFRs in TNBC, only minimal success has been achieved due to resistance mechanisms [35]. In this study, we demonstrated that triazole derived estradiol analogs directly target the epidermal growth factor receptor (EGFR) by suppressing its phosphorylation at the docking site Y1068 in a concentration-dependent manner, followed by inhibition of its downstream PI3K/AKT/mTOR and MAPK signaling pathways in MDA-MB-231 (Figure 6 and Figure 7). Phosphorylation of EGFR on Y1068 creates binding sites for the adaptor protein, Grb2, leading to activation of the MAPK/ERK cascade, and a binding site for Gab1, which recruits the p85 subunit of phosphatidylinositol 3-kinase (PI3K), leading to AKT activation [39]. Treating MDA-MB-231 cells with Fz25, Fz57 and Fz200 doses resulted in decreased expressions of total EGFR and phosphorylated EGFR (Y1068) in a dose-dependent manner within 24 h. Furthermore, the MAPK pathway was inactivated as the compounds downregulated the expression levels of phosphorylated-ERK1/2 (T202/Y204) and -ARaf (Ser299) in a dose-dependent fashion. Similarly, the PI3K/AKT/mTOR pathway was also deactivated as the expression levels of activated proteins, phospho-mTOR (Ser2448), phosphor-Akt (Ser473) and phosphor-p70S6a (Ser411) were suppressed when the TNBC model was exposed to Fz25, Fz57 and Fz200 doses. These findings support our previous results [15] where we showed that estrone analogs with cucurbitacin pharmacophores downregulated EGFR signaling and concomitantly suppressed PI3K/AKT/mTOR and MAPK signaling in MDA-MB-468 TNBC cells. Even though different generations of estrone analogs are tested against TNBC cells with varying levels of EGFR expressions, both studies arrive at similar conclusions.

Furthermore, we assessed the triazole-estradiol hybrids’ ability to halt cell division as another proliferation mechanism that occurs in all cells, including TNBC cells. The cell cycle is one of the major regulatory mechanisms of cell growth, and abnormal regulation of the cell cycle is a notable hallmark of cancer cells [40]. Cyclin D1, an important controller of cell cycle progression, can turn extracellular mitotic signals into DNA synthesis by binding to cyclin-dependent kinase 4/6 (CDK4/6), which in turn, inactivate the E2F inhibitor, the retinoblastoma protein (RB), thus accelerating the cell cycle switch from G_1_ to S phase [40,41]. Dyrk1B (dual-specificity tyrosine-regulated kinase 1B), a checkpoint kinase, is important for G_1_- to S-phase cell cycle transition and is responsible for modulating the expression of several cell cycle activators and regulators [42]. Dyrk1B maintains cells in quiescent G_0_ state by phosphorylating cyclin D1 (cell division activator), p^21^ and p^27^ (cell cycle regulators), thereby serving as a cell cycle check point to block G_1_- to S-phase transitions [43]. Cyclin E is another crucial regulator of the cell cycle responsible for cells entry into S phase and acts in association with CDK2. Cyclin D1, cyclin E, and Dyrk1B are overexpressed in TNBC [43,44,45], and these shorten the G_1_ phase of the cell cycle and mediate cells quick entry into the S phase [41]. Cell cycle analysis revealed that Fz25 and Fz200 inhibited cell growth by arresting G_0_/G_1_ phase of cell division whereas Fz57 arrested cells in S phase (Figure 5). These results agree with our previous findings where we showed that estrone analogs with modified cucurbitacin pharmacophores arrested MDA-MB-468 TNBC cells in G_0_/G_1_ phase in vitro [15]. However, these findings contrast reports by Nolte et al. 2023, where they reveal that estradiol analogs, 2-ethyl-3-O-sulphamoyl-estra-1,3,5(10)16-tetraene and tetrahydroisoquinoline-based 2-methoxy estradiol caused the accumulation of MDA-MB-231 breast cancer cells in the G_2_/M phase due to depolymerization of the microtubules [46]. We further investigated the impact of triazole-estradiol hybrids on key cell cycle activators and regulators within G_1_ to S phase. Fz25 downregulated the expression levels of Cyclin D1 and Dyrk1B whereas Fz57 and F200 only suppressed the expression levels of cyclin E in a dose-dependent fashion, alluding to their halting of cell division in different phases. These findings are comparable to our past reports where estrone analogs with modified cucurbitacin pharmacophores suppressed cyclins D1, E and Dyrk1B expressions in different cancers [14]. These outcomes reveal that different generations of estrone analogs can alter key regulators and activators of the cell cycle in different phases in various cancers.

Apoptosis induction is one of the strategies to stop the proliferation of cancer cells and stimulate their death. Misregulation in apoptosis mechanisms relate to several diseases, in particular cancer. The apoptosis mechanism can be initiated through two different pathways based on the nature and origin of the stimuli. These include the intrinsic (death receptor) and the extrinsic (mitochondrial) pathways. The intrinsic pathway transmits signals of internal cellular damage to the mitochondrion, which loses its structural integrity, and forms an apoptosome that initiates the caspase cascade. The extrinsic pathway to apoptosis leads through death ligands and death receptors to the activation of the caspase cascade, which results in proteolytic degradation of the cell architecture. We tested triazole-derived estradiol analog apoptosis induction in MDA-MB-231 cells using different assays, including annexin V, chromatin condensation and protein markers involved in the mitochondrial apoptosis cascade. Viable cells contain phosphatidylserine (PS) located on the inside of the cell membrane. When apoptosis occurs, PS flip will occur. PS moves to the outside surface of cells to which annexin V binds. Analyzing MDA-MB-231 treated cells with real-time annexin V assay, it was observed that Fz25 and Fz57 induced PS translocation within 6 h in a dose-dependent fashion when compared to camptothecin, positive control (Figure 8). However, Fz200 induced PS translocation within 48 h in a dose-dependent fashion when compared to camptothecin, (Figure 8). Subsequently, morphological changes including chromatin condensation induced by the estradiol analogs were analyzed by fluorescence microscopy after staining cells with Hoechst 33342. Chromatin condensation paralleled by DNA fragmentation is one of the most important criteria used to identify apoptotic cells. Fz25, Fz57 and Fz200 clearly induced chromatin condensation within 24 h of drug treatment (Figure 9). These results are in line with studies where different generations of estrone analogs induced PS translocation and chromatin condensation in MDA-MB-231 cells [30,31]. Furthermore, the triazole-estradiol analog effects on markers involved in the mitochondrial apoptosis pathway were investigated. Bcl-2, an anti-apoptotic protein, binds to various proapoptotic family members and inhibits their insertion into the mitochondrial membrane. Upon receiving an apoptotic signal, Bcl-2 releases proapoptotic proteins, allowing them to form a complex on the mitochondrial membrane. Cytochrome c is consequently released into the cytoplasm, triggering increased caspase activity which leads to cell death. In apoptotic cells, cytochrome c binds to Apaf-1 to induce apoptosome formation. The Apaf-1 apoptosome catalyzes the autocatalytic activation of the caspase-9 zymogen (initiator caspase), which subsequently cleaves and activates caspase-3 [47]. Finally, caspase-3 cleaves and inactivates PARP (poly (ADP-ribose) polymerase) which is important for damaged DNA repair [48]. Dosing Fz25 against MDA-MB-231 cells resulted in no effect on Bcl-2 (Figure 10A) but Fz57 and Fz200 significantly decreased the expression levels of Bcl-2 (Figure 10B,C) which is indicative of mitochondrial apoptosis. In addition, Fz25, Fz57, and Fz200 administrations to MDA-MB-231 cells resulted in mitochondrial apoptosis induction where increased expressions of cytochrome c and Apaf-1 were observed (Figure 10A–C). Moreover, dosing triazole-estradiol congeners to MDA-MB-231 cells resulted in slightly decreased expressions of PARP 1 (Figure 11A–C), in contrast to the increased expressions of cleaved PARP 1 in a dose-dependent manner (Figure 11A–C). Likewise, caspases-9 and -3 were downregulated slightly after triazole-estradiol analog administration (Figure 11). These reports are comparable to our previous report where we showed that estrone analogs with cucurbitacin pharmacophores induced mitochondrial apoptosis in EGFR-dependent NSCLC and TNBC [14,15]. Nonetheless, this study is the first to report on triazole-derived estradiol analogs targeting mitochondrial-induced apoptosis in an MDA-MB-231 TNBC model.

To qualify to be a drug candidate, some prerequisites such as physicochemical traits should be attained by any chemical entity. The absorption, distribution, metabolism, excretion, and toxicity (ADMET) properties of a molecule are important factors in successful drug development [49]. In silico analysis was performed to confirm the reliability of in vitro anticancer screenings. Utilizing physicochemical properties that were compiled from the SwissADME and ADMETLab 2.0 webservers, the triazole-estradiol analog drug-likeness was assessed. As illustrated in Table S1, the majority of estradiol analogs comply with Veber’s and Lipinski’s rule criteria (with a maximum of two rule violations). However, it should be noted that many of the most successful drugs do not fit these guidelines [50]. The TPSA of all analogs were less than 140 Å^2^ and acceptable, showing that the molecules will have medium to high gastrointestinal absorption (GIA) properties. Drug candidates exhibit poor absorption when their TPSA is higher than 140 Å^2^, which is benchmarked for marketed drugs [51]. TPSA has a positive correlation with mass and the molecules with a mass higher than 500 g/mol were observed to have TPSA beyond the range of 0–140. GIA and blood–brain barrier (BBB) permeability (Table S2) are important properties of a drug that is intended for widespread use. There have been initial studies to improve GI permeation of molecules for orally delivered drugs that are poorly absorbed [52]. Triazole-estradiol congeners demonstrated acceptable GIA and crossed the BBB. The MlogP and LogD_7.4_ were ≤5 (Table S1) and are deemed to be appropriated for “drug like” small molecules with a molecular weight below 500 amu [53]. High lipophilicity (MlogP and LogD_7.4_) usually results in a high risk of metabolic clearance, metabolite-related toxicity, and promiscuity-related off-target toxicity, and low lipophilicity might promote renal clearance [54]. The Abbott bioavailability score of greater than zero (Table S2) indicates that the estradiol analogs have a substantial bioavailability and cross the cell membrane efficiently [55]. Thus, the triazole-estradiol analogs demonstrated adequate drug-likeness and physicochemical characteristics indicating their potential as therapeutic medication.

In silico pharmacokinetic/ADME evaluations were executed, and the data are presented in Table S2, which unveils that all the screened compounds except Fz57 and Fz100 showed acceptable Caco-2 permeability (recommended value > −5.15) corresponding to appropriate MDCK permeability (values should be > 2 × 10^−6^ cm/s). All compounds had a proper volume of distribution (VD) as all were within the predicted VD range of 0.04–20 L/kg. Also, most compounds, except Fz100, Fz600 and Fz550, showed high plasma protein binding (PPB), value < 95% and concomitantly posed a low % fraction of unbound drug (predicted value > 5%). These parameters predict that triazole-estradiol analogs can exhibit lower non-specific tissue targeting due to their lower propensity to distribute into all organ tissues and therefore hopefully show higher specific binding to target tissues. All molecules disclosed moderate clearance (within range of 5–15 mL/min/kg) and acceptable half-life less than 3 h.

Permeability glycoprotein (P-gp) influences the ADMET properties of many xenobiotics (foreign drugs or chemicals), and it is important to investigate the interaction between the transporter protein and the hit molecule. It limits the cellular uptake and metabolism of compounds by acting as a unidirectional efflux pump to extrude its substrate from inside to outside of cells. From Table S3, only Fz25, like sorafenib, was neither substrate nor inhibitor of P-glycoprotein (*p* value < 0.3), demonstrating its desirable properties as a potential therapeutic agent. Inhibitors block the metabolic activity of CYP450 enzymes while inducers increase CYP450 enzyme activity by increasing enzyme synthesis. Hence, caution should be taken when prescribing a drug known to be a CYP450 inhibitor or inducer. All triazole-estradiol analogs were strong inhibitors (*p* value > 0.3) of CY1A2, CYP2C19, CYP2C9, CYP2D6 and CYP3A4 enzymes. Therefore, the target compounds may need additional optimization, or the dose adjusted to account for a potential decrease or increase in metabolism.

In silico human hepatotoxicity, drug-induced liver injury, AMES toxicity, carcinogenicity, and eye irritation were assessed, and are presented in Table S4. All the synthesized compounds were not eye irritants. All compounds except Fz25 were carcinogenic, AMES toxic, induced liver injury and were hepatotoxic (*p* value > 0.3). Furthermore, few compounds including Fz57, Fz514 and Fz516 were hERG blockers (*p* value > 0.9). Overall, Fz25 was sufficiently safe and warrants additional investigation as a novel anti-TNBC agent.

**Figure 13:**
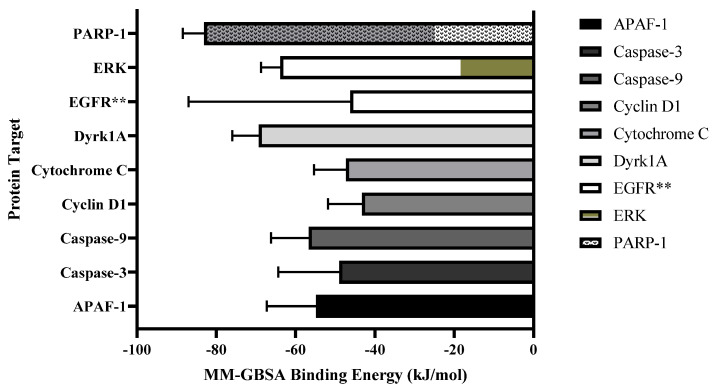
MMGBSA binding energy results for binding of Fz25 to proteins. Binding energies are calculated as an average over the course of the simulation. ** Note the large standard deviation is due to flexibility of the EGFR protein arm.

To explore the structure–activity relationships of these analogs for further analog design, we performed molecular dynamic simulations to determine the importance of various functional groups in hit compound Fz25. Figure 13 outlines the binding of Fz25 in both EGFR and ERK, two proteins that showed inhibition in a dose-dependent response. Ultimately, it was determined that the C_3_ benzyl group was important for binding, a fact corroborated by the cytotoxicity data in the MDA-MB-231 cell line, i.e., all benzylic species exhibit cytotoxicity. Similarly, we saw the importance of the triazole-estradiol core for binding of Fz25 to ERK, where both aromatic groups show importance for cation-π interactions. Because of these interactions, we are currently developing a second-round analog design focusing on modulating the triazole side chain of analogs with benzylic functionality. We ultimately wish to also modify the structure of Fz25, our lead compound, by functionalizing the steroid core itself. Nonetheless these initial stages of design and evaluation suggest the modification of these analogs is warranted and needed.

## 5. Conclusions

The success of previously synthesized triazole-estradiol analogs has prompted their evaluation in new areas of cancer biology. Due to the lack of a reliable option for treatment in triple negative breast cancer, we tested these triazole analogs in two triple-negative cell lines, MDA-MB-231 and MDA-MB-468. We elucidated that hit analogs Fz25, Fz57 and Fz200 showed the best potency for MDA-MB-231, ultimately inhibiting cell growth in the G_1_ phase of the cell cycle and reducing the activity of the cell-cycle mediators cyclin-D_1_, cyclin E and Dyrk1B. Additionally, Fz25, Fz57 and Fz200 were able to inhibit EGFR and EGFR-dependent downstream mediators mTOR and ERK in a dose-dependent response. Fz25, Fz57 and Fz200 were also able to induce the initial stages of apoptosis, including altering the morphology of cells, as well as modulating key proteins in apoptosis, such as APAF-1 and caspase enzymes. Finally, molecular modeling elucidated the key pharmacophores of these molecules necessary for binding, of which some are currently being used for the design of second-round analogs. Future aspects of this work include evaluation of second-round analogs, additional optimization of hit compounds, as well as testing of analogs with in vivo studies.

## Figures and Tables

**Table 1:** Cytotoxic effects of triazole-estradiol analogs against triple negative breast cancer cells. In vitro cytotoxic activities (IC_50_, μM) of estradiol analogs against breast cancer cells. IC_50_ values were calculated by non-linear regression analysis. Values represent mean ± SD of the quadruplicate experiment (*n* = 4).

	IC_50_ (µM)	
Compound	MDA-MB-231	MDA-MB-468
Sorafenib	10.62 ± 0.02	28.38 ± 3.13
Fz25	8.12 ± 0.85	25.43 ± 3.68
Fz57	21.18 ± 0.23	>50
Fz60	>50	>50
Fz100	>50	>50
Fz200	10.86 ± 0.69	>50
Fz313	>50	>50
Fz514	>50	>50
Fz516	>50	>50
Fz518	>50	>50
Fz550	50.00 ± 0.20	>50
Fz552	>50	45.36 ± 2.87
Fz600	>50	>50
Estrone	80.07 ± 0.85	>100

## Data Availability

Data are available upon request.

## Supplementary Materials

The following supporting information can be downloaded at: https://www.mdpi.com/article/10.3390/molecules30030605/s1, Table S1: Lipinski’s rule of five and Veber’s rule for drug likeness; Table S2: Calculated physicochemical properties of triazole-estradiol analogs; Table S3: Interactions of analogs with P-glycoprotein and cytochrome P450 isoenzymes; Table S4: Predicted toxicity profiles of triazole-estradiol analogs; Figure S1: Effect of G1- or S- phase cell cycle regulators after treatment of MDA-MBA-231 cells with triazole-estradiol analogs; Figure S2: Effect of triazole-estradiol analogs treatment on EGFR and downstream ERK1/2 effector molecules in MDA-MB-231 cells; Figure S3: Effect of triazole-estradiol analogs treatment on Akt pathway proteins in MDA-MB-231 cells; Figure S4: Expression levels of cytosolic cytochrome C and APAF1 in MDA-MB-231 cells; Figure S5: In-Cell Western quantification of apoptosis-associated markers.

## Abbreviations

TNBC—Triple negative breast cancer, EGFR—Epidermal growth factor receptor, ERK—Extracellular related kinase, mTOR—Mechanistic target of rapamycin, APAF—Apoptotic protease activating factor, PARP—Poly (ADP-ribose) polymerase, ADMET—Adsorption, distribution, metabolism, extraction, and toxicity, RAS—Rat sarcoma virus, PI3K—Phosphoinositide-3-kinase, MTT—3-(4,5-dimethylthiazol-2-yl)-2,5-diphenyltetrazolium bromide, DMSO—Dimethyl sulfoxide.
