# Supplementary material for: Triazole-Estradiol Analogs Induce Apoptosis and Inhibit EGFR and Its Downstream Pathways in Triple Negative Breast Cancer
Source: Molecules. 2025 Jan 30;30(3):605. doi: 10.3390/molecules30030605 (PMC11820259; doi:10.3390/molecules30030605)
Supplement: Supplementary file 1 [file molecules-30-00605-s001.zip › molecules-3377826-supplementary.pdf]

Supplemental Information

Triazole-Estradiol Analogs Induce Apoptosis and Inhibit EGFR and its Downstream Pathways in Triple Negative Breast Cancer

Felix Acheampong*[1], Trevor Ostlund*[2], Emily Hedge[2], Jacqueline Laddusaw[2], Faez Alotaibi[2,3], Yaseen A.M.M. Elshaier[2,4], Fathi Halaweish[2]

[1]*Department of Preclinical Pharmacology and Toxicology, Verve Therapeutics Inc., Boston, MA 02215, USA.*

[2]*Department of Chemistry and Biochemistry, College of Natural Sciences, South Dakota State University, Brookings, SD 57007, USA.*

[3]*Department of chemistry, college of Science, Qassim University, Buraydah, Saudi Arabia*

[4]*Department of Organic and Medicinal Chemistry, University of Sadat City, Monufia, Egypt.*

*These authors contributed equally to this work

Correspondence: Prof. Fathi Halaweish, Fathi.Halaweish@sdstate.edu, Department of Chemistry and Biochemistry, South Dakota State University, Brookings, SD 57007, USA.

Table of Contents:

S2: **Table S1.** Lipinski's rule of five and Veber's rule for drug-likeness.

S3: **Table S2**. Calculated physicochemical properties of triazole-estradiol analogs.

S4: **Table S3.** Interactions of analogs with P-glycoprotein and cytochrome P450 isoenzymes.

S5: **Table S4**. Predicted toxicity profiles of triazole-estradiol analogs.

S6: Figure S1. Effect of G_1- or S- phase cell cycle regulators after treatment of MDA-MB-231 cells with triazole-estradiol analogs.

S6: Figure S2. Effect of triazole-estradiol analogs treatment on EGFR and downstream ERK1/2 effector molecules in MDA-MB-231 cells.

S7: Figure S3. Effect of triazole-estradiol analogs treatment on Akt pathway proteins in MDA-MB-231 cells.

S7: Figure S4. Expression levels of cytosolic cytochrome C and APAF1 in MDA-MB-231 cells.

S8: Figure S5. In-Cell Western quantification of apoptosis-associated markers.

Table S1. Lipinski's rule of five and Veber's rule for drug-likeness analysis of triazole-estradiol analogs.

Compound	MW (g/mol)	MLogP	LogD7.4	HBD	HBA	TPSA (Å2)	ROTB	Number of Lipinsk's Rule Violations	Number of Veber Rule Violations	PAINS Alerts	% ABS†
Sorafenib	464.82	2.91	3.66	3	7	92.35	9	0	0	0	77.1
Fz 25	429.24	3.88	4.49	2	5	71.03	4	0	0	0	84.5
Fz 57	562.29	4.19	4.85	2	7	89.27	8	2	0	0	78.2
Fz 60	458.55	3.07	4.06	3	5	100.27	5	0	0	0	74.4
Fz 100	452.55	1.84	2.63	2	6	100.71	4	0	0	0	74.3
Fz 200	500.59	3.42	3.84	2	6	106.34	7	1	0	0	72.3
Fz 313	486.61	3.46	4.30	3	7	100.27	5	0	0	0	74.4
Fz 514	538.64	4.25	4.10	1	6	99.24	6	2	0	0	74.8
Fz 516	510.63	4.11	4.51	1	5	82.17	5	2	0	0	80.7
Fz 518	496.60	3.92	4.32	2	5	93.17	4	0	0	0	76.9
Fz 550	506.64	3.37	3.37	1	6	97.55	6	1	0	0	75.3
Fz 552	478.63	3.23	3.84	1	5	80.48	5	0	0	0	81.2
Fz 600	464.28	3.03	3.53	2	5	91.48	4	0	0	0	77.4

Lipinski's rule of five and Golden Triangle rule for drug-likeness analysis of Triazole estrone analogs. TPSA topological polar surface area, ROTB number of rotatable bonds.

†%ABS = 109 − [0.345 × TPSA]

Table S2. Calculated physicochemical properties of triazole-estradiol analogs.

Compound	Caco-2 Permeability (Log Units of cm/s)	MDCK Permeability (cm/s)	GI Absorption	Abbot Bioavailability Score	% Plasma Protein Binding (PPB)	Volume Distribution (VD) (L/kg)	BBB Penetration P-value	% Fraction Unbound (Fu)	Clearance (CL) (mL/min/kg)	Half-Life ($T_{1/2}$) (h)
Sorafenib	-5.338	1.000E-05	Low	0.001	97.810	0.845	0.523	1.880	5.490	0.095
Fz25	-4.850	1.800E-05	High	0.010	97.470	1.429	0.403	1.101	12.465	0.057
Fz57	-5.216	1.700E-05	0.004	0.625	98.940	1.171	0.353	0.527	12.161	0.026
Fz60	-4.880	1.800E-05	0.006	0.715	96.130	0.946	0.666	1.559	14.699	0.131
Fz100	-5.257	1.900E-05	0.037	0.990	64.310	1.959	0.728	23.030	11.079	0.105
Fz200	-4.924	2.600E-05	0.006	0.961	95.820	0.389	0.769	2.032	6.820	0.071
Fz313	-4.846	1.900E-05	0.004	0.581	96.670	0.873	0.589	1.536	14.555	0.097
Fz514	-5.032	2.700E-05	0.004	0.985	93.630	0.449	0.795	4.062	4.629	0.021
Fz516	-4.913	2.400E-05	0.004	0.869	96.000	0.924	0.581	1.127	9.297	0.013
Fz518	-4.908	2.100E-05	0.005	0.842	95.050	0.985	0.635	1.772	11.729	0.033
Fz550	-5.029	3.500E-05	0.016	0.995	82.190	0.612	0.661	9.957	5.417	0.031
Fz552	-4.961	3.300E-05	0.012	0.970	92.720	0.887	0.533	2.221	9.898	0.019
Fz600	-5.150	3.000E-05	0.104	0.987	89.400	1.030	0.631	6.262	13.129	0.048

Table S3. Interactions of triazole-estradiol analogs with P-glycoprotein and cytochrome P450 isoenzymes.

Compound	Pgp-substrate (P-value)	Pgp-inhibitor (P-value)	CYP1A2 inhibitor (P-value)	CYP2C19 inhibitor (P-value)	CYP2C9 inhibitor (P-value)	CYP2D6 inhibitor (P-value)	CYP3A4 inhibitor (P-value)
Sorafenib	No (0.053)	No (0.048)	Yes (0.517)	Yes (0.944)	Yes (0.923)	Yes (0.654)	Yes (0.723)
Fz25	No (0.125)	No (0.058)	Yes (0.863)	Yes (0.964)	Yes (0.919)	Yes (0.667)	Yes (0.927)
Fz57	No (0.031)	Yes (0.995)	No (0.210)	Yes (0.907)	Yes (0.962)	No (0.413)	Yes (0.936)
Fz60	Yes (0.794)	No (0.069)	Yes (0.580)	Yes (0.922)	Yes (0.878)	Yes (0.699)	Yes (0.893)
Fz100	Yes (0.591)	No (0.035)	No (0.030)	No (0.151)	No (0.122)	No (0.015)	No (0.460)
Fz200	No (0.025)	Yes (0.963)	No (0.427)	Yes (0.898)	Yes (0.881)	Yes (0.566)	Yes (0.836)
Fz313	Yes (0.947)	No (0.127)	No (0.313)	Yes (0.910)	Yes (0.881)	Yes (0.659)	Yes (0.924)
Fz514	No (0.002)	Yes (0.922)	Yes (0.642)	Yes (0.871)	Yes (0.915)	No (0.342)	Yes (0.915)
Fz516	No (0.002)	Yes (0.883)	Yes (0.534)	Yes (0.903)	Yes (0.940)	Yes (0.507)	Yes (0.963)
Fz518	No (0.036)	No (0.067)	Yes (0.787)	Yes (0.945)	Yes (0.924)	Yes (0.660)	Yes (0.938)
Fz550	No (0.100)	Yes (0.800)	No (0.079)	No (0.488)	No (0.450)	No (0.186)	Yes (0.837)
Fz552	No (0.048)	Yes (0.656)	No (0.087)	Yes (0.637)	No (0.492)	No (0.404)	Yes (0.935)
Fz600	Yes (0.641)	Yes (0.536)	No (0.087)	No (0.492)	No (0.446)	No (0.113)	Yes (0.872)

Table S4. Predicted Toxicity profiles of triazole-estradiol analogs

Compound	hERG Blockers[ǂ]	Human Hepatotoxicity (P-value)	Drug Induced Liver Injury (P-value)	AMES Toxicity (P-value)	Carcinogenicity (P-value)	Eye Irritation (P-value)
Sorafenib	No (0.765)	Yes (0.912)	Yes (0.967)	Yes (0.540)	No (0.331)	No (0.010)
Fz 25	No (0.871)	No (0.264)	No (0.089)	No (0.328)	No (0.188)	No (0.016)
Fz 57	Yes (0.980)	No (0.302)	Yes (0.907)	Yes (0.810)	Yes (0.824)	No (0.009)
Fz 60	No (0.787)	No (0.428)	Yes (0.636)	No (0.155)	Yes (0.942)	No (0.011)
Fz 100	No (0.212)	Yes (0.544)	No (0.126)	No (0.044)	Yes (0.986)	No (0.010)
Fz 200	No (0.876)	No (0.334)	Yes (0.613)	No (0.204)	Yes (0.934)	No (0.010)
Fz 313	No (0.856)	No (0.416)	Yes (0.632)	No (0.193)	Yes (0.953)	No (0.011)
Fz 514	Yes (0.932)	No (0.235)	Yes (0.751)	Yes (0.099)	Yes (0.940)	No (0.007)
Fz 516	Yes (0.963)	No (0.348)	Yes (0.692)	No (0.179)	Yes (0.945)	No (0.008)
Fz 518	No (0.889)	No (0.348)	Yes (0.648)	No (0.156)	Yes (0.945)	No (0.008)
Fz 550	No (0.677)	Yes (0.591)	No (0.339)	No (0.015)	Yes (0.973)	No (0.010)
Fz 552	No (0.855)	Yes (0.555)	No (0.293)	No (0.014)	Yes (0.981)	No (0.010)
Fz 600	No (0.534)	Yes (0.545)	No (0.178)	No (0.025)	Yes (0.981)	No (0.011)

[ǂ] Molecules with P value > 0.9 are considered extremely toxic.

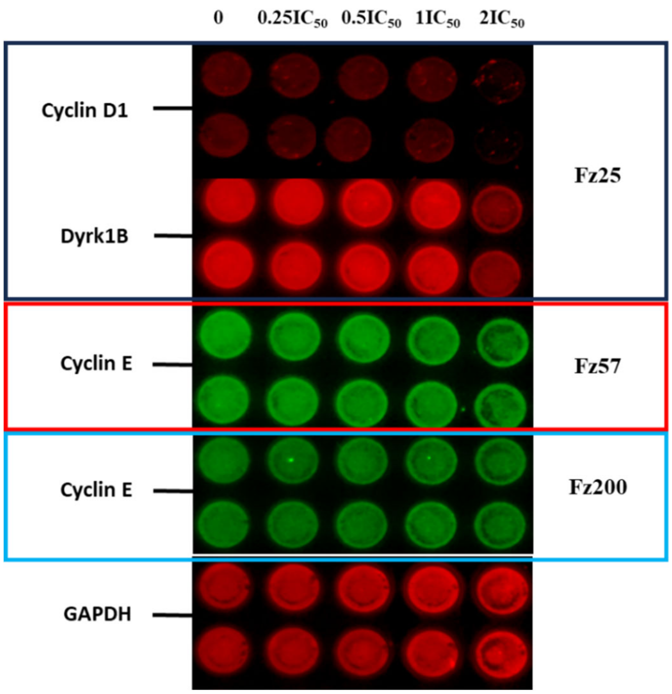

Figure S1. Effects on G1- or S-phase cell cycle regulators after treatment of MDA-MB-231 cells with estradiol analogs. Scanned images from Fiji software, and expression levels of the proteins normalized to GAPDH.

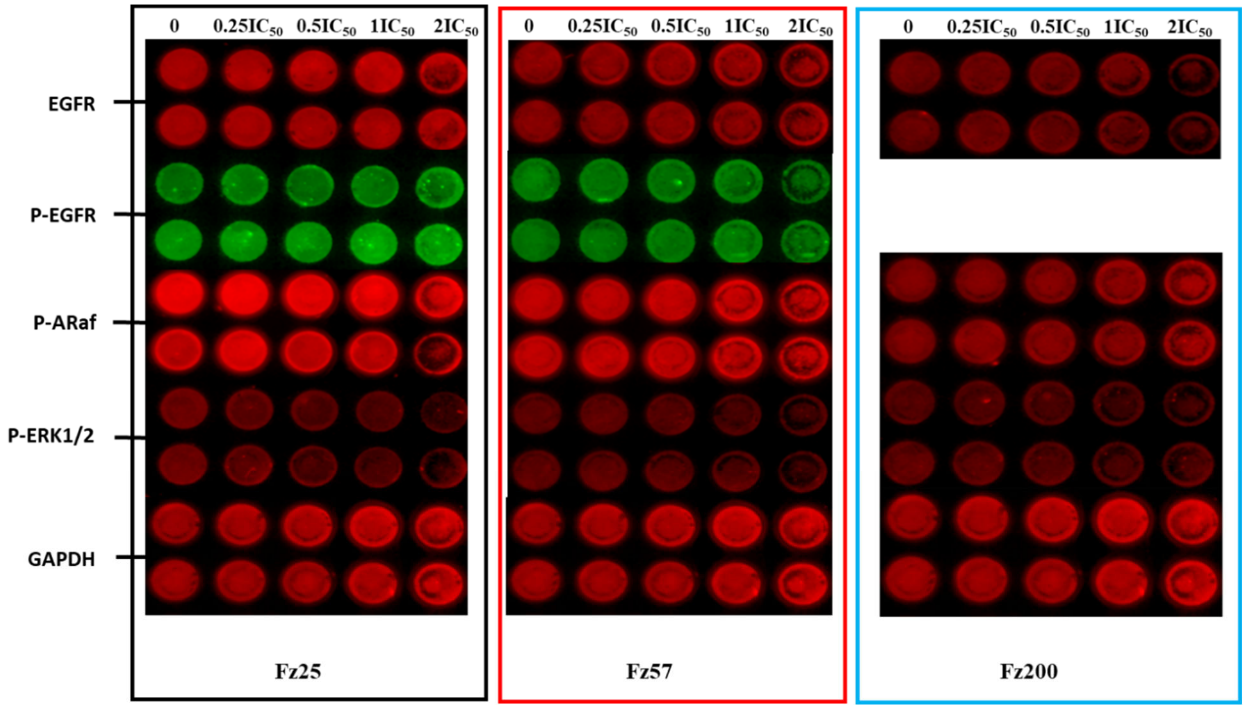

Figure S2. Effect of triazole-estradiol analogs treatment on EGFR and downstream ERK1/2 effector molecules in MDA-MB-231 cells. Scanned images from Fiji software, and expression levels of the proteins normalized to GAPDH.

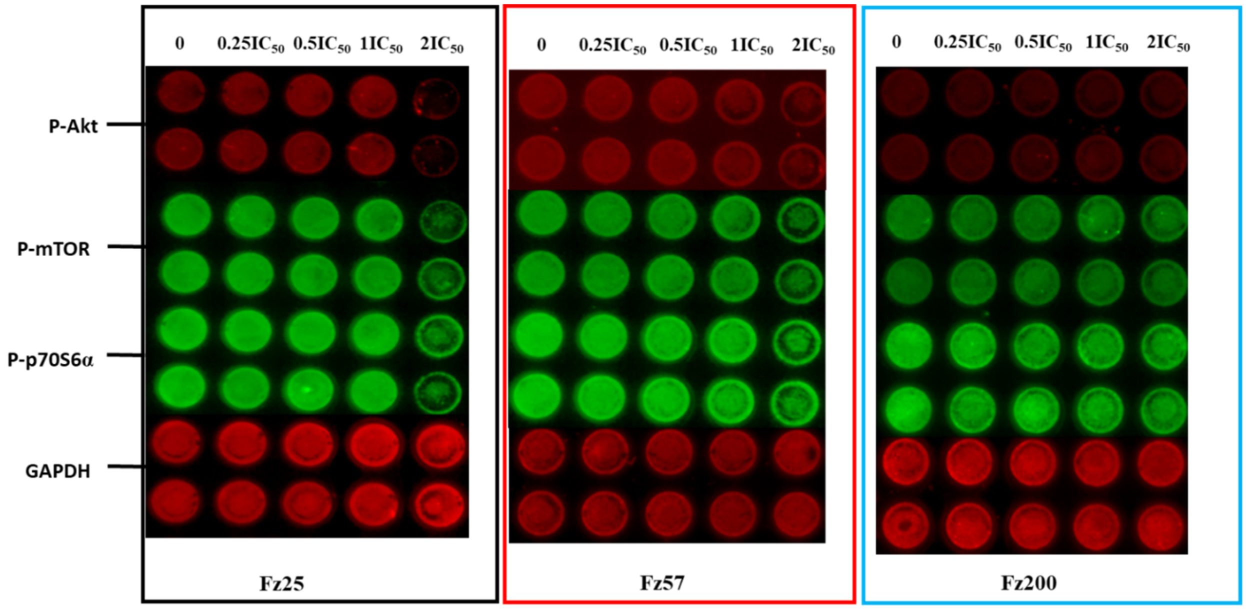

Figure S3. Effect of triazole-estradiol analogs treatment on Akt pathway proteins in MDA-MB-231 cells. Scanned images from Fiji software, and expression levels of the proteins normalized to GAPDH.

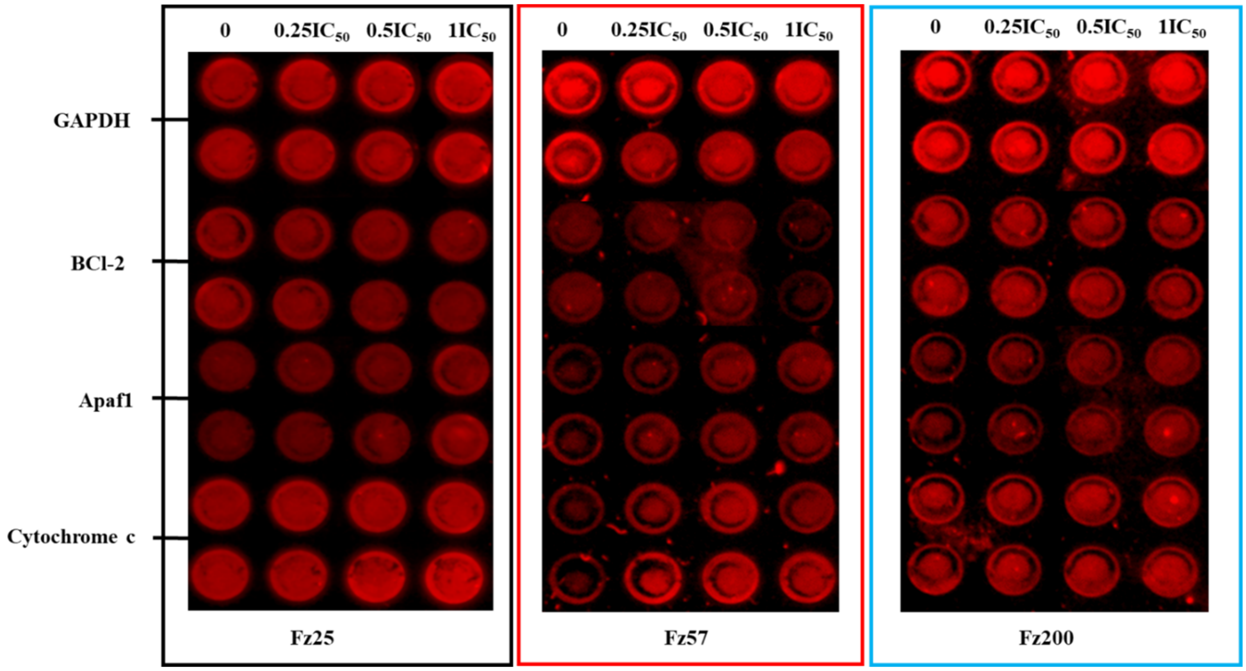

Figure S4. Expression levels of cytosolic cytochrome C and APAF1 in MDA-MB-231 cells assayed by In-Cell Western (ICW). Scanned images from Fiji software, and expression levels of BCl-2, APAF1 and cytochrome C normalized to GAPDH.

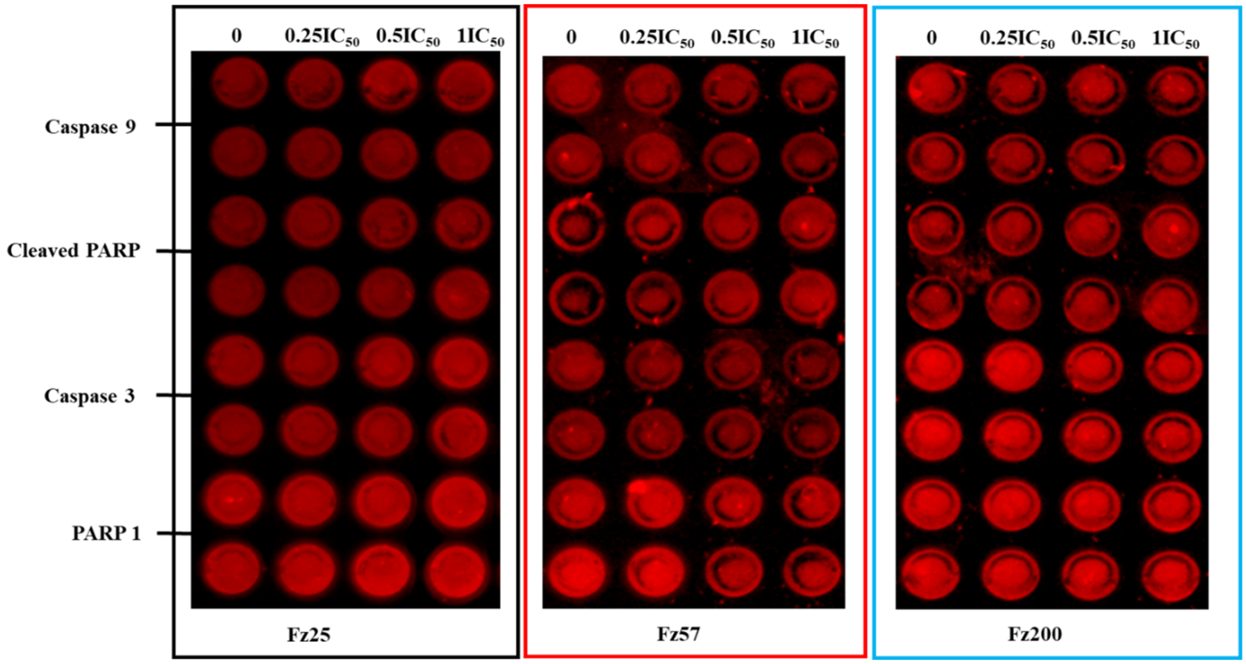

Figure S5. In-Cell Western quantification of apoptosis-associated markers. Scanned images from Fiji software, and expression levels of the proteins normalized to GAPDH. GAPDH in figure 10 was used for normalization as all images were acquired in the same experiment.
